# 2-aminoethoxydiphenyl borate provides an anti-oxidative effect and mediates cardioprotection during ischemia reperfusion in mice

**DOI:** 10.1371/journal.pone.0189948

**Published:** 2017-12-21

**Authors:** Hirofumi Morihara, Masanori Obana, Shota Tanaka, Ikki Kawakatsu, Daisuke Tsuchiyama, Shota Mori, Hiroshi Suizu, Akiko Ishida, Rumi Kimura, Izuru Tsuchimochi, Makiko Maeda, Takehiko Yoshimitsu, Yasushi Fujio, Hiroyuki Nakayama

**Affiliations:** 1 Laboratory of Clinical Science and Biomedicine, Graduate School of Pharmaceutical Sciences, Osaka University, Suita, Osaka, Japan; 2 Laboratory of Synthetic Medicinal Chemistry, Graduate School of Pharmaceutical Sciences, Osaka University, Suita, Osaka, Japan; 3 Educational and Research Unit of Pharm.D. Graduate School of Pharmaceutical Sciences, Osaka University, Suita, Osaka, Japan; 4 Laboratory of Synthetic Organic and Medicinal Chemistry, Division of Pharmaceutical Sciences, Graduate School of Medicine, Dentistry, and Pharmaceutical Sciences, Okayama University, Okayama, Japan; Emory University, UNITED STATES

## Abstract

Excessive levels of reactive oxygen species (ROS) and impaired Ca^2+^ homeostasis play central roles in the development of multiple cardiac pathologies, including cell death during ischemia-reperfusion (I/R) injury. In several organs, treatment with 2-aminoethoxydiphenyl borate (2-APB) was shown to have protective effects, generally believed to be due to Ca^2+^ channel inhibition. However, the mechanism of 2-APB-induced cardioprotection has not been fully investigated. Herein we investigated the protective effects of 2-APB treatment against cardiac pathogenesis and deciphered the underlying mechanisms. In neonatal rat cardiomyocytes, treatment with 2-APB was shown to prevent hydrogen peroxide (H_2_O_2_) -induced cell death by inhibiting the increase in intracellular Ca^2+^ levels. However, no 2-APB-sensitive channel blocker inhibited H_2_O_2_-induced cell death and a direct reaction between 2-APB and H_2_O_2_ was detected by ^1^H-NMR, suggesting that 2-APB chemically scavenges extracellular ROS and provides cytoprotection. In a mouse I/R model, treatment with 2-APB led to a considerable reduction in the infarct size after I/R, which was accompanied by the reduction in ROS levels and neutrophil infiltration, indicating that the anti-oxidative properties of 2-APB plays an important role in the prevention of I/R injury *in vivo* as well. Taken together, present results indicate that 2-APB treatment induces cardioprotection and prevents ROS-induced cardiomyocyte death, at least partially, by the direct scavenging of extracellular ROS. Therefore, administration of 2-APB may represent a promising therapeutic strategy for the treatment of ROS-related cardiac pathology including I/R injury.

## Introduction

Cardiovascular diseases (CVDs) are the leading cause of death and disability worldwide. One of the most dominant causes of death by CVD is myocardial infarction (MI), which is accompanied by massive cardiomyocyte death. While recent progress in clinical cardiology led to a successful reduction of MI size by reperfusion therapies, the size-limiting effects are substantially lessened by ischemia-reperfusion (I/R) injury, due to the cellular damage caused by the restoration of coronary blood flow [1]. Therefore, novel therapeutic approaches for preventing myocardial I/R are required.

Whereas the mechanisms underlying I/R injury and myocardial protection have been intensively investigated during the last three decades [2, 3], the clinical efficacy of few pharmacological therapies have been demonstrated [4]. Two major intracellular second messengers, Ca^2+^ and reactive oxygen species (ROS), are known to play dominant roles in cardiac I/R injury [5, 6]. In the first several minutes of myocardial reperfusion, a burst of oxidative stress is induced from a variety of sources [7] [8], and leads to apoptotic and non-apoptotic cell death [9]. Animal model research indicated that the overproduction of ROS may be responsible for I/R injury [10, 11], but the results of clinical studies with the administration of antioxidant therapy at the onset of myocardial reperfusion have been mixed [12]. Therefore, further mechanisms underlying cardiac I/R or novel tools for the regulation of ROS should be investigated.

2-aminoethoxydiphenyl borate (2-APB) is a membrane-permeant, lipophilic compound that was originally reported as a blocker of intracellular inositol 1,4,5-trisphosphate receptor (IP_3_R) [13, 14]. Currently, increasing evidence indicates that 2-APB has inhibitory effects on multiple ion channels such as transient receptor potential (TRP) family of cation channels, potassium channels [15], and volume-regulated anion channels [16]. Multiple members of TRP channels were shown to be sensitive to 2-APB, including TRPC3, TRPC5, TRPC6, TRPM2, TRPM3, and TRPM7 [17–22]. Therefore, the major biological function of 2-APB is generally considered to be based on channel inhibitory effect.

Reduced I/R injury by 2-APB treatment has been reported in multiple organs with promising results [23–25]. Therefore, we hypothesized that 2-APB may exert a protective effect during cardiac I/R injury through a channel inhibitory effect and assessed the effects of 2-APB administration on ROS-induced cardiomyocyte death or cardiac I/R injury in mice. Unexpectedly, we found that these protective effects are based on direct ROS inhibition, not channel blocking effect, in cardiomyocytes. The obtained results indicate that ROS scavenging by 2-APB may be a promising therapeutic approach in cardiac I/R injury.

## Materials and methods

### Animal care

The care of all animals complied with the Osaka University animal care guidelines. All experimental procedures conformed to the Guide for the Care and Use of Laboratory Animals, promulgated by the US National Institutes of Health, and were approved by Animal Care and Use Committee in Graduate School of Pharmaceutical Sciences, Osaka University (approved as Douyaku 26–4, the animal experimental protocol number 26–4 of Graduate School of Pharmaceutical Sciences). All animal experiments were in accordance with the Guide for the Care and Use of Laboratory Animals, Eighth Edition, updated by the US National Research Council Committee in 2011. A total of 108 male mice underwent surgical procedures in this study, and all experimental protocols were completed within 24 h following the surgery. All efforts were made to minimize suffering and distress of the animals. Humane endpoints were used for all mice involved in the study and once animals reached endpoint criteria including cachexic condition evaluated by body weight loss (more than 10%), loss of voluntary motion and failure to react to stimuli, they are euthanized by inhalation of isoflurane in a euthanasia chamber as soon as possible. Death of the animals was confirmed by monitoring the absence of breath after the removal of the carcass from the euthanasia chamber. No animals died before meeting criteria for euthanasia except those that died of causes related to surgical procedures, such as bleeding.

### Reagents

Reagents used in this study included H_2_O_2_ (31 wt%; Santoku Chemical Industries Co, Japan), BAPTA-AM (Merck Millipore, Billerica, MA), xestospongin C (Wako Pure Chemical Industries, Japan), SKF-96365 (Focus Biomolecules, Plymouth Meeting, PA), mefenamic acid (Tokyo Chemical Industry Co, Japan), 2-APB, AA-861 and phenylephrine (Sigma-Aldrich, St. Louis, MO).

### Neonatal rat cardiomyocyte (NRCM) isolation

Neonatal rats were purchased from Kiwa Laboratory Animals Co.Ltd. NRCMs were isolated and cultured as previously described [26]. In brief, hearts excised from neonatal rats were minced and digested with a solution containing 0.1% collagenase type IV (Sigma-Aldrich, St. Louis, MO) and 0.1% trypsin (Thermo Fisher Scientific, Waltham, MA) to obtain a suspension of single cells. After incubating the cells in a culture dish for 60–90 min at 37°C in 5% CO_2_ atmosphere, floating cells were collected as NRCMs. Isolated NRCMs were cultured in Dulbecco’s Modified Eagles Medium (DMEM; Sigma-Aldrich, St. Louis, MO) containing high glucose (4.5 g/L) with L-glutamine (4 mM) and sodium bicarbonate (3.7 g/L), supplemented with 10% fetal bovine serum (FBS) and bromodeoxyuridine (0.1 μg/mL; Sigma-Aldrich, St. Louis, MO).

### Cell viability assays

The isolated NRCMs were cultured in a serum-free medium for 24 h and were pretreated with 2-APB, specific 1,4,5-Inositol trisphosphate receptor blocker (xestospongin C), TRP channel blocker (SKF-96365), TRPM7 blocker (AA-861) or TRPM3 blocker (mefenamic acid), 1 h before the addition of H_2_O_2_. An equal volume of vehicles (i.e., ethanol for xestospongin C and DMSO for SKF-96365, AA-861, mefenamic acid) was added as a control 1 h before the addition of H_2_O_2_. Four hours after the addition of H_2_O_2_, cell viability was measured using the CellTiter Blue assay kit (Promega, Madison, WI), according to the manufacturer’s protocol. The assay is based on the ability of living cells to convert a redox dye (resazurin) into a fluorescent product (resorufin). The cells in 96-well culture plate were incubated with 20 μL of CellTiter Blue reagent for 3 h at 37°C in a humidified, 5% CO_2_ atmosphere. The fluorescence was measured using a 96-well plate reader (SpectraMAX M5e, excitation at 560 nm and emission at 590 nm; Molecular Devices, Sunnyvale, CA)

### Intracellular calcium measurements

To elucidate the underlying mechanism of Ca^2+^ chelator mediated inhibition in H_2_O_2_-induced cardiomyocyte death, intracellular calcium levels were monitored as previously reported with modifications [27]. NRCMs were cultured on a flexiPERM disc (Sarstedt, Germany) using DMEM with 10% FBS. The cells were washed with Hank’s balanced salt solution (HBSS) containing 1.26 CaCl_2_, 0.493 MgCl_2_, 0.407 MgSO_4_, 5.33 KCl, 0.441 KH_2_PO_4_, 4.17 NaHCO_3_, 137.93 NaCl, 0.338 Na_2_HPO_4_ and 5.56 D-glucose (in mM) and incubated in 10 μM Fura 2-AM (Sarstedt, Germany) /HBSS working solution for 1 h. Subsequently, cells were washed with HBSS three times, which was followed with the incubation for 30 min, and the resulting fluorescence was detected by AQUA COSMOS (Hamamatsu Photonics, Japan) monitoring the excitation spectra at 340 or 380 nm with fixed emission at 505 nm. The fluorescence ratio of 340/380 nm excitation was calculated to assess intracellular Ca^2+^ concentration.

### Intracellular ROS measurements

Intracellular ROS levels were determined using the fluorescent marker 2′,7′-dichlorofluorescin diacetate (DCFH-DA, D6883; Sigma-Aldrich, St. Louis, MO), according to the manufacturer’s protocol. Briefly, NRCMs were cultured in 96-well culture plate and incubated with 5 μM DCFH-DA for 30 min at 37°C, and they were washed twice with the phenol red-free DMEM (Sigma-Aldrich, St. Louis, MO). After the pretreatment with/without 2-APB for 1 h, the cells were stimulated with 100 μM H_2_O_2_ or 2 mM phenylephrine (PE) and fluorescence intensity was measured using a 96-well plate reader (SpectraMAX M5e, excitation at 485 nm and emission at 590 nm; Molecular Devices, Sunnyvale, CA) to assess the increase in the intracellular ROS levels. Based on DCF fluorescent intensity levels, the ROS increase ratio was calculated as difference from values of those at 0 min and represented as %Fluorescence increase which is normalized to those at 60 min of vehicle-treated control.

### ^1^H-NMR measurements and thin-layer chromatography

To examine the direct interaction between 2-APB and H_2_O_2_, ^1^H-NMR measurement and thin-layer chromatography were performed [28] [29]. Five milligrams of 2-APB (0.0222 mmol) were dissolved in methanol-*d*_4_ (0.6 mL) in an NMR tube at room temperature, and the mixture was subjected to ^1^H-NMR analysis to obtain an authentic spectrum (spectrum A). H_2_O_2_ (31% w/v, 25 μL, 0.222 mmol) was then added to this mixture. After shaking for a few seconds, the mixture was subjected to the ^1^H-NMR analysis and the second spectrum was recorded in 5 min (spectrum B). After 60 min, the mixture was analyzed to obtain the third spectrum (spectrum C). All ^1^H-NMR spectra were recorded on a 300 MHz JEOL AL-300 spectrometer in the deuterated solvent, and the chemical shifts were reported with a residual peak of CD_2_HOD as an internal standard. The reaction products were further examined by thin-layer chromatography (TLC) using TLC plates (TLC silica gel 60 F_254_; Merck Millipore, Billerica, MA).

### I/R model and 2-APB treatment in mice

Murine I/R model was generated following the protocol which we have established previously [30]. Briefly, C57BL/6 male mice (7–9 weeks old; Japan SLC) were anesthetized using 1.5% isoflurane (Pfizer, New York City, NY) carried in 100% oxygen followed by the intubation, and they were ventilated with 100% oxygen containing 1.5% isoflurane. After left-side thoracotomy, 7–0 silk suture was tied around the left coronary artery with a slipknot, which can be released by pulling one end coming out from the body. Infarction was confirmed by the discoloration of the ventricle. The chest and the skin were closed with 5–0 silk sutures. Sham operation was performed as a control. The mice were revived during the 30-min ischemic period, and anesthetized shortly at the end of ischemia to release the slipknots, and the heart was allowed to reperfuse for 24 h. Using this experimental protocol, 27 out of 32 mice survived after the surgical procedure and the mortality was minimized to less than 20%. Twenty-four hours after the reperfusion, the mice were euthanized and the slipknot was retied. Evans blue (5%; Wako Pure Chemical Industries, Japan) was injected into the left ventricle (LV), and the hearts were removed. Isolated hearts were sectioned, and viable myocardium was stained with 2% triphenyl tetrazolium chloride (Nacalai Tesque, Japan), as described previously [30]. Myocardial area not at risk, area at risk (AAR), and infarcted area (IA) were quantified using the ImageJ Analysis software (National Institutes of Health). In the 2-APB group, 10 mg/kg of 2-APB were intravenously administered immediately before the reperfusion (indicated concentrations in 125 μL of saline / 25 g of body weight), whereas the control group received the same volume of saline containing vehicle over the same period. There was no difference in mortality between two groups.

### Quantitative RT-PCR

To assess the expression levels of proinflammatory cytokines that are involved in the recruitment of the inflammatory cells that produce ROS, quantitative RT-PCR was performed according to the manufacturer’s instructions. Hearts were obtained from mice that underwent 30 min ischemia followed by reperfusion with the period of 30 min or 6 h. Using this protocol, 31 out of 40 mice survived after the surgical procedure. Total RNA was isolated from hearts after I/R using QIAzol reagent (QIAGEN, Germantown, MD). First-strand cDNA was synthesized from 1 μg of total RNA with oligo (dT) primers and then used to determine the expression of the genes of interest. The mRNA expression for *il-6*, *il-1β* and *tnf-α* in the LVs from the ligation point to the apex was quantified by real-time RT-PCR using the Applied Biosystems StepOne Real-Time PCR systems with the SYBR Green system (Applied Biosystems, Carlsbad, CA). As an internal control, the expression of *Gapdh* was estimated using the SYBR Green system. Primer sequences are displayed in S1 Table.

### Immunohistochemical analyses

For the immunohistochemical analysis, hearts were obtained from mice that underwent 30 min ischemic procedure followed by 24 h reperfusion. Hearts were embedded in O.C.T. Compound (Sakura Finetech, Japan), frozen in liquid nitrogen, and cut into 5 μm-thick sections using Leica CM1950 (Leica Biosystems, Germany). Immunohistochemical analyses were performed using the indirect immune-peroxidase method. After the inhibition of endogenous peroxidase activity, the sections were incubated overnight followed by incubation with the primary antibody such as anti-Ly-6G for neutrophils (Gr-1) (1:100) (clone 1A8; BioLegend, San Diego, CA). An appropriate immunoglobulin-conjugated peroxidase-labeled polymer amino acid (anti-rat IgG; Vector Laboratories, Burlingame, CA) was used as a secondary antibody. After visualization with 3,3’-diaminobenzidine, the sections were mounted with Entellan™ New (Merck Millipore, Billerica, MA). Staining was examined using FSX100 Inverted Microscope (Olympus, Hicksville, NY). The number of Gr-1-positive cells in the AAR was counted in five random fields in three nonconsecutive sections per heart by a researcher who was blinded to the experimental conditions and displayed as a number per mm^2^.

### Dihydroethidium (DHE) fluorescence analysis

DHE fluorescence analysis was performed to examine the generation of superoxide according to a previous report [30]. In brief, frozen sections (5 μm-thick) were prepared from hearts exposed to ischemia for 30 min and reperfused for 24 h, and stained with 10 μM DHE (Sigma-Aldrich, St. Louis, MO) in Krebs-HEPES buffer composed of (all in mM) 99.01 NaCl, 4.69 KCl, 1.87 CaCl_2_, 1.20 MgSO_4_, 1.03 K_2_HPO_4_, 25.0 NaHCO_3_, 20.0 Na-HEPES, and 11.1 glucose (pH 7.4) at 37°C for 30 min in dark. The intensities of fluorescence were quantitfied using ImageJ Analysis software (National Institutes of Health) by a researcher who was blinded to the assay conditions.

### Oxidative stress biomarker determination *in vivo*

To corroborate the involvement of ROS in 2-APB-induced cardioprotection *in vivo* further, oxidative stress levels were assessed in mice. The levels of diacron reactive oxygen metabolites (d-ROMs) and biological anti-oxidant potential (BAP) were measured using a free radical analyzer system (FREE Carrio Duo; Diacron International, Italy), as previously reported [31]. All analyses were performed using animal serum samples collected via inferior vena cava of mice exposed to ischemia for 30 min and reperfused for 30 min or 6 h. Using this experimental protocol, 19 out of 24 mice survived after the I/R surgical procedure. We chose those timepoints as an immediate period after reperfusion or a beginning phase of neutrophil infiltration. The d-ROMs test shows the amount of organic hydroperoxides, which corresponds to 0.8 mg/L of H_2_O_2_ per unit. The BAP test provides an estimate of the global anti-oxidant capacity of serum, by measuring the reduced ferric ion levels. BAP/d-ROM ratio was determined as a marker of relative anti-oxidant capacity.

### Statistical analysis

Data are presented as mean ± standard error of mean (SEM). Comparisons between the two groups were performed with Student’s t-test. One-way analysis of variance (ANOVA) followed by Tukey-Kramer test was used for multiple comparisons. Differences were considered statistically significant when the calculated (two-tailed) *P* value was <0.05.

## Results

### Treatment with 2-APB suppresses ROS-induced cell death and intracellular Ca^2+^ increase in NRCMs

**Fig 1:**
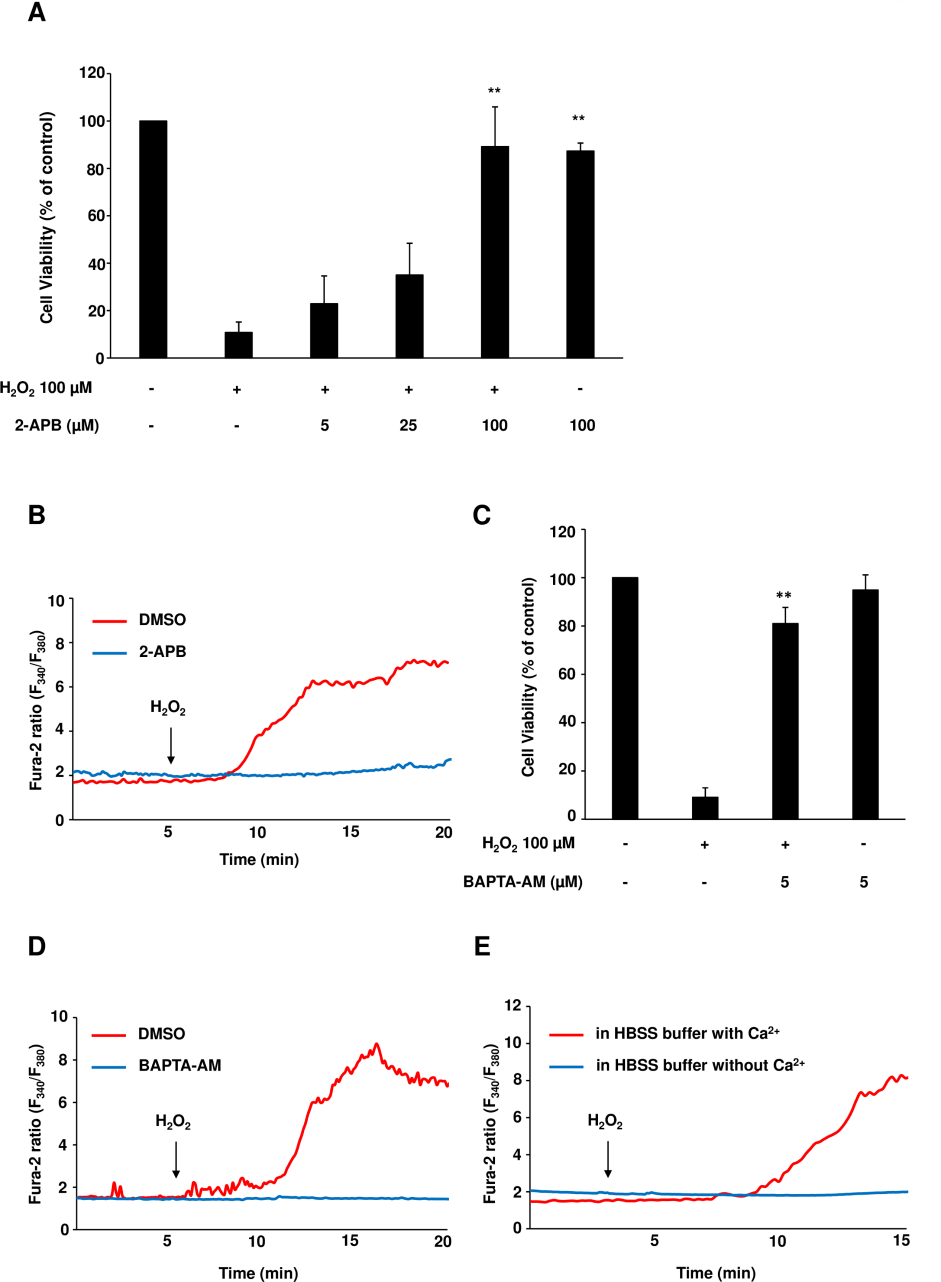
Treatment with 2-APB attenuated H_2_O_2_-induced cell death and Ca^2+^ influx in cardiomyocytes. (A) Analysis of the viability of neonatal rat cardiomyocytes (NRCMs) with or without 100 μM 2-APB pretreatment for 1 h, followed by subsequent stimulation with 100 μM H_2_O_2_ for 4 h. Results are presented as mean ± SEM obtained in five to seven independent experiments. (B) Representative fura-2 ratios from NRCMs pretreated with or without 2-APB for 1 h, followed by stimulation with H_2_O_2_. (C) NRCMs were pretreated or not with 5 μM BAPTA-AM, which was followed by H_2_O_2_ treatment and the degree of cell survival is presented as mean values ± SEM obtained in six or seven independent experiments. (D) Representative fura-2 ratios from NRCMs pretreated or not with 10 μM BAPTA-AM for 1 h, followed by H_2_O_2_ treatment. (E) Representative fura-2 ratios obtained using NRCMs treated with 100 μM H_2_O_2_ in HBSS buffer with or without Ca^2+^ prepared by the administration of EDTA. **P<0.01, compared with H_2_O_2_-treated samples, using one-way ANOVA.

First, we examined the effects of 2-APB treatment on ROS-induced cell death in NRCMs. Stimulation with H_2_O_2_ induced massive cell death 4 h following the administration, which was considerably decreased by pretreatment with 2-APB (Fig 1A). Treatment with 2-APB alone failed to induce cell death at a dose of 100 μM. Cell viability assay demonstrated that the pretreatment with 2-APB 1 h prior to H_2_O_2_ stimulation attenuated H_2_O_2_-induced cell death in a dose-dependent manner and showed almost complete inhibition of cell death at 100 μM (cell death, 10.8±4.4% in pretreated *vs*. 89.2±16.7% in non-pretreated samples; Fig 1A). Since previous reports suggested that H_2_O_2_-induced cell death is associated with the impaired Ca^2+^ homeostasis in other cell types [32], we assessed intracellular Ca^2+^ concentrations during H_2_O_2_ treatment with or without 2-APB pretreatment in NRCMs. A considerable increase in intracellular Ca^2+^ concentration was observed within 10 min after H_2_O_2_ treatment, which was completely abolished by 2-APB pretreatment (Fig 1B). To elucidate the relationship between the 2-APB-mediated cell death inhibition and the prevention of an increase in the intracellular Ca^2+^ levels, we used BAPTA-AM, an intracellular Ca^2+^ chelator, to analyze whether the reduction of Ca^2+^ levels attenuates cardiomyocyte death following the H_2_O_2_ treatment. Pretreatment with a low dose of BAPTA-AM significantly attenuated H_2_O_2_-induced cardiomyocyte death (9.1±3.9% *vs*. 81.0±6.7%; Fig 1C). In addition, pretreatment with BAPTA-AM prevented the increase in Ca^2+^ concentration after H_2_O_2_ stimulation (Fig 1D). Finally, to determine whether the increase of Ca^2+^ levels is due to the extracellular Ca^2+^ entry, EDTA was used for the removal of the extracellular Ca^2+^, and intracellular Ca^2+^ levels were assessed during H_2_O_2_ treatment. The extracellular chelation of Ca^2+^ using EDTA-containing medium was shown to prevent an increase in Ca^2+^ levels after H_2_O_2_ stimulation (Fig 1F). Taken together, these results indicate that 2-APB treatment attenuates H_2_O_2_-induced cell death through the inhibition of increase of intracellular Ca^2+^ levels.

### 2-APB-sensitive Ca^2+^ channels are not involved in H_2_O_2_-induced cardiomyocyte death

**Fig 2:**
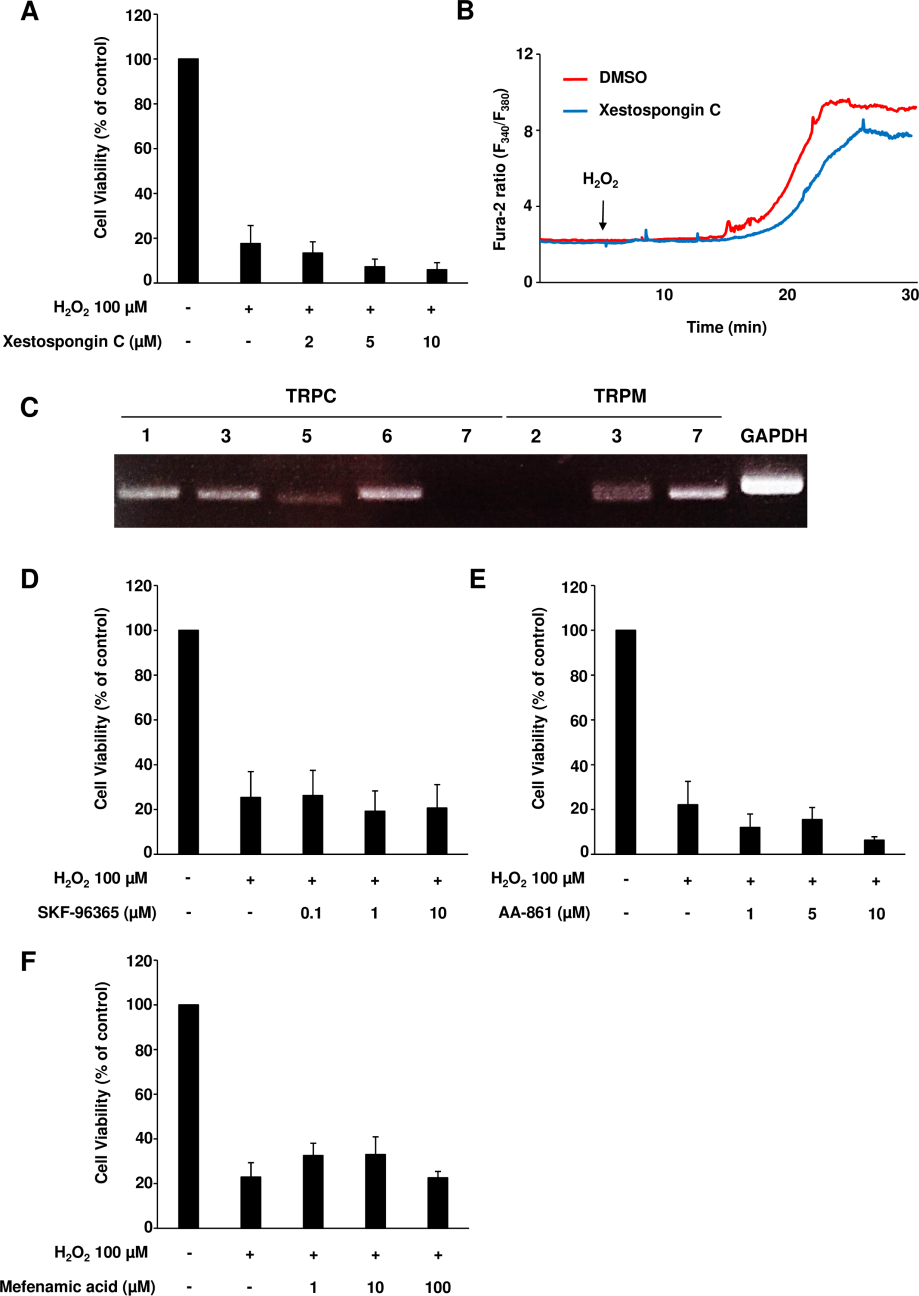
Specific inhibition of 2-APB-sensitive Ca^2+^ channels failed to prevent H_2_O_2_-induced cardiomyocyte death. (A) Neonatal rat cardiomyocytes (NRCMs) were pretreated or not with xestospongin C, a specific IP_3_R inhibitor, for 1 hour, which was followed by H_2_O_2_ treatment. Cell viability levels were determined. Results are shown as mean values ± SEM from three independent experiments. (B) Representative fura-2 ratio obtained using NRCMs pretreated or not with xestospongin C, followed by H_2_O_2_ treatment. (C) The expression levels of 2-APB-sensitive TRP channels in NRCMs were analyzed by RT-PCR. A representative image with a 40-cycle amplification is shown. (D-F) NRCMs were pretreated or not with SKF-96365 (D, a TRPC inhibitor), AA-861 (E, a TRPM7 inhibitor) and mefenamic acid (F, a TRPM3 inhibitor), followed by H_2_O_2_ treatment. Cell viability rate is presented. Results are presented as mean values ± SEM obtained in four independent experiments.

We investigated whether IP_3_R was involved in H_2_O_2_-induced cardiomyocyte death, since 2-APB was reported to be an IP_3_R antagonist [33]. Pretreatment with xestospongin C, a specific IP_3_R inhibitor, failed to suppress H_2_O_2_-induced cardiomyocyte death (Fig 2A). Consistently, NRCMs pretreated with xestospongin C displayed a similar increase in intracellular Ca^2+^ levels compared to those in the H_2_O_2_-treated control (Fig 2B), suggesting that IP_3_R is not involved in 2-APB-mediated cytoprotection.

Multiple TRP channels were shown to be suppressed by 2-APB treatment [34, 35]. To date, multiple TRPCs and TRPM channels, such as TRPM2, TRPM3 and TRPM7 have been reported to be 2-APB sensitive [17, 20, 21]. We analyzed these channels using RT-PCR, and observed that TRPC1, 3, 5 and 6, as well as TRPM3 and 7 are expressed in NRCMs (Fig 2C). The inhibition of TRPCs by SKF-96365, TRPM3 by mefenamic acid, and TRPM7 by AA-861 failed to prevent H_2_O_2_-induced cell death (Fig 2D–2F), although the doses used were sufficient to inhibit the targeted channels based on the previously reported studies [36]. Pretreatment with SKF-96365 at a dose of 10 μM, which is twice higher compared to a previously reported one, was enough to inhibit capacitative calcium entry in NRCMs failed to prevent cell death, suggesting those TRPCs are not involved in H_2_O_2_-induced cardiomyocyte death (Fig 2D). Likewise, pretreatment with AA-861 which blocks TRPM7 activity at 10 μM in several previous reports [37, 38] failed to inhibit cell death with a similar dose (Fig 2E). Moreover, pretreatment with mefenamic acid, a specific inhibitor of TRPM3 whose EC_50_ is reported to be approximately 6 μM [39], also failed to attenuate cell death at 1, 10 and 100 μM (Fig 2F). Collectively, those results indicate that major 2-APB sensitive Ca^2+^ channels are not involved in the presented model of H_2_O_2_-induced cardiomyocyte death.

### 2-APB reacts with and scavenges extracellular ROS

**Fig 3:**
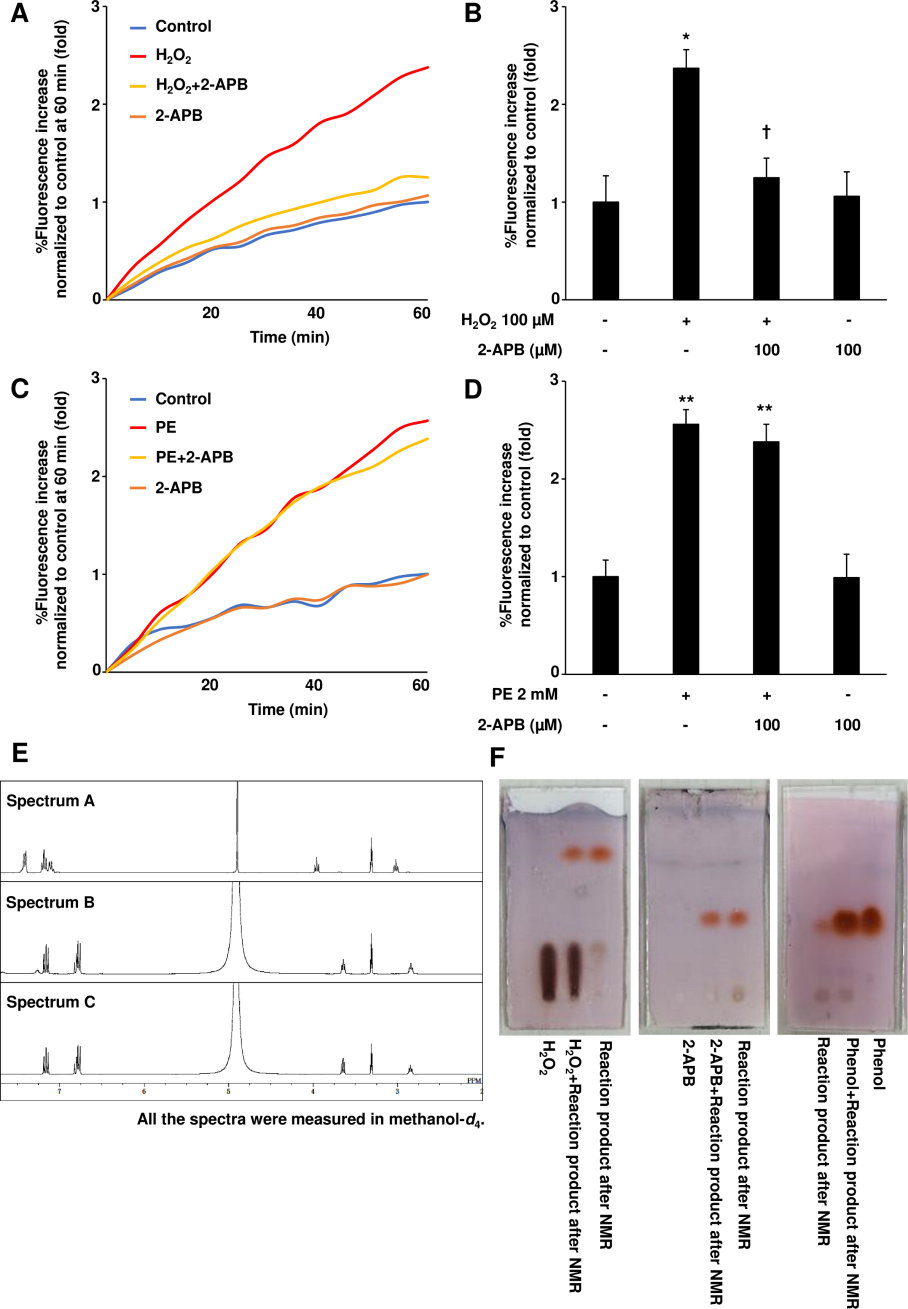
Hydrogen peroxide directly reacts with 2-APB. (A) Neonatal rat cardiomyocytes (NRCMs) were loaded with 5 μM DCF, and treated with 2-APB and H_2_O_2_. Average results of %Fluorescence increase were obtained from three independent experiments. (B) Intracellular ROS levels at 60 min after H_2_O_2_ stimulation were estimated from DCF fluorescence levels in NRCMs pretreated or not with 2-APB. Results of %Fluorescence increase are presented as mean values ± SEM obtained in three independent experiments. *P<0.05 *vs*. untreated control, ^†^*P*<0.05 *vs*. H_2_O_2_ treated samples, obtained using one-way ANOVA. (C) NRCMs were loaded with 5 μM DCF and treated with 2-APB for 1 h, followed by stimulation with 2 mM PE. Average results of %Fluorescence increase were obtained from three independent experiments. (D) Intracellular ROS levels at 60 min after PE stimulation, estimated using DCF fluorescence intensity levels in NRCMs pretreated or not with 2-APB. Results of %Fluorescence increase are presented as mean values ± SEM obtained in three independent experiments. **P<0.01 *vs*. untreated control, using one-way ANOVA. (E) Representative ^1^H-NMR spectra obtained from 2-APB (spectrum A) or 2-APB in direct reaction with H_2_O_2_ for 5 min (spectrum B) or 60 min (spectrum C) are shown. (F) Representative thin-layer chromatography (TLC) images are presented. Reaction product indicates samples obtained from the reaction between 2-APB with H_2_O_2_ used for NMR measurement. Comparison between the reaction products and authentic H_2_O_2_ (left), 2-APB (middle), or phenol (right) is presented.

To decipher the mechanism underlying 2-APB-mediated myocyte protection, we examined ROS inhibition by this compound. We assessed intracellular ROS levels in NRCMs and observed considerable increase in these levels after H_2_O_2_ stimulation compared with those in the control, which are abolished after 2-APB pretreatment (Fig 3A). We demonstrated that 2-APB treatment led to a significant reduction in the H_2_O_2_-induced intracellular ROS increase 60 min after the treatment (DCF intensity, 87.0±7.0% *vs*. 45.8±7.6%; Fig 3B), suggesting that 2-APB directly inhibits ROS generation. In contrast to this, 2-APB treatment failed to inhibit a phenylephrine (PE)-induced increase in the intracellular ROS levels, which occurs through NADH/NADPH oxidase activation [40] (Fig 3C and 3D). Furthermore, pre-treatment with *N*-acetylcysteine (NAC), reported to inhibit G-protein coupled receptor-induced ROS level increase [41], prevented ROS level increase after PE administration (S1 and S2 Figs. These results indicate that 2-APB treatment does not inhibit an increase in intracellular ROS levels, but that it directly reacts with extracellular ROS, resulting in the inhibition of the ROS toxic effects in NRCMs. The direct reaction between 2-APB and H_2_O_2_ was examined by ^1^H-NMR. The ^1^H-NMR spectra of 2-APB (spectrum B and C) recorded at 5 and 60 min, respectively, after the addition of 10 molar equivalents of H_2_O_2_ to 2-APB, were shown to change significantly from the authentic spectrum (spectrum A). This observation suggests that 2-APB chemically reacts with H_2_O_2_ (Fig 3E). TLC analysis showed that a vermillion spot identical to that of an authentic phenol appeared on a TLC plate by the treatment of 2-APB with H_2_O_2_, supporting that the oxidation of 2-APB with H_2_O_2_ generates phenol (Fig 3F). Taken together, these results indicate that 2-APB has a potential to scavenge extracellular ROS and functions as an anti-oxidative compound.

### Administration of 2-APB attenuates myocardial damage after I/R in mice

**Fig 4:**
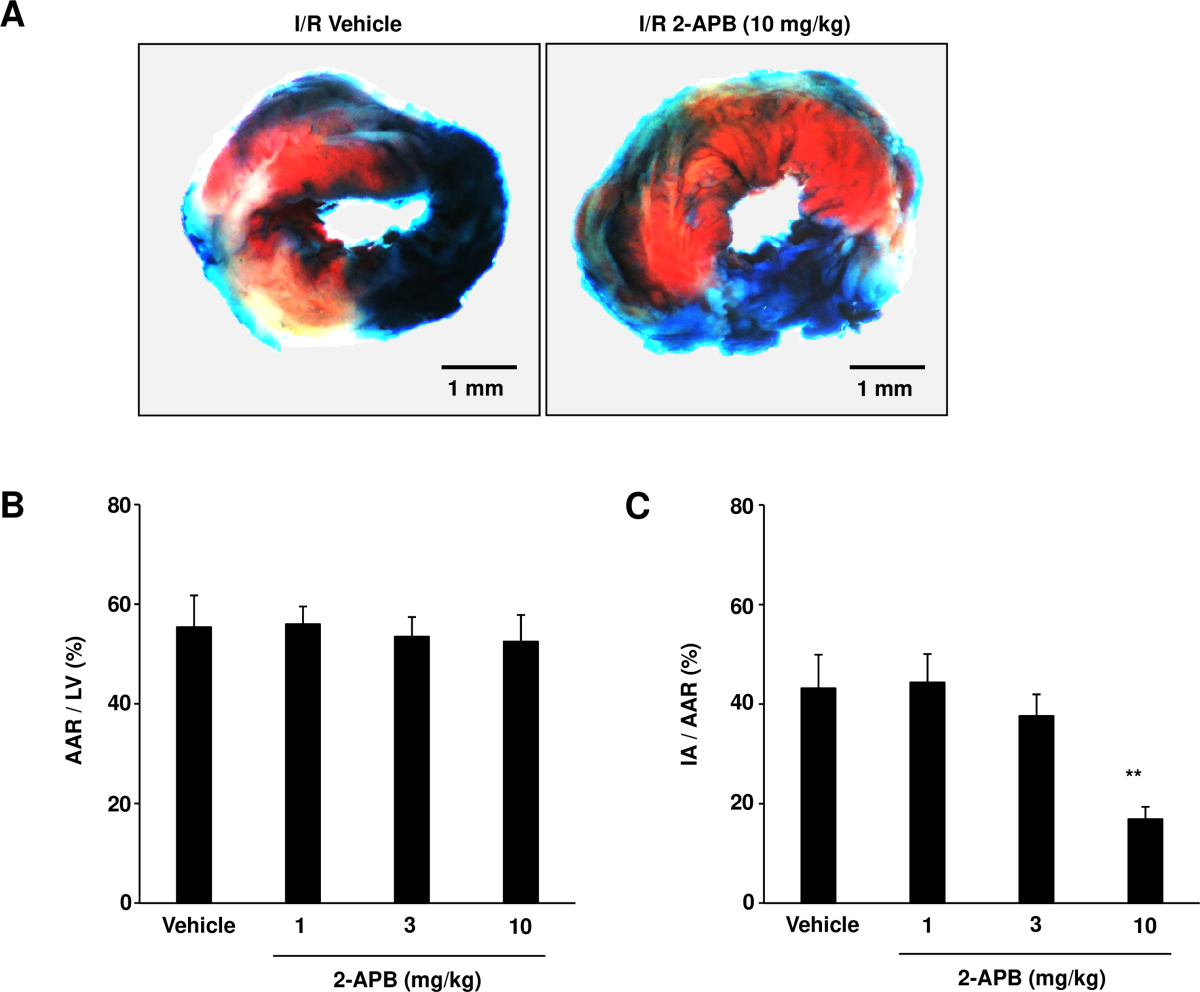
Single 2-APB administration attenuates cardiac I/R injury. C57BL/6 mice were exposed to 30 min ischemia, followed by 24 h reperfusion, and treated with 2-APB. Areas at risk (AAR) were estimated by exclusion of area stained with Evans blue, while myocardial infarct areas (IAs) were detected by 2% triphenyl tetrazolium chloride (TTC) staining. (A) Representative images are shown. Scale bar, 1 mm. (B and C) AAR normalized to LV (B) values and IA size normalized to AAR (C) were quantified. Results are presented as mean values ± SEM (43.2±6.8% *vs*. 16.9±2.5% in IA/AAR, n = 10 mice for vehicle; n = 4 mice for 1 mg/kg of 2-APB; n = 5 mice for 3 mg/kg of 2-APB; n = 8 mice for 10 mg/kg of 2-APB). **P<0.01 vs. vehicle-treated control, obtained using one-way ANOVA.

To examine the effects of 2-APB on cardiac injury *in vivo*, C57BL/6 mice were randomized to either 2-APB- or vehicle-treated group, and subjected to left coronary artery occlusion for 30 min followed by 24 h reperfusion. Vehicle or 2-APB was administered via intravenous bolus injection immediately before the reperfusion. We demonstrated that 2-APB treatment failed to affect the area at risk (AAR) (Fig 4A and 4B). However, the ratio between IA and AAR was shown to decrease significantly after a single injection of 2-APB (Fig 4A and 4C). In contrast, a single dose NAC treatment failed to attenuate cardiac I/R injury (S3 and S4 Figs), suggesting that 2-APB has unique anti-oxidant properties.

### 2-APB treatment attenuates ROS activity and inflammatory responses during I/R injury

**Fig 5:**
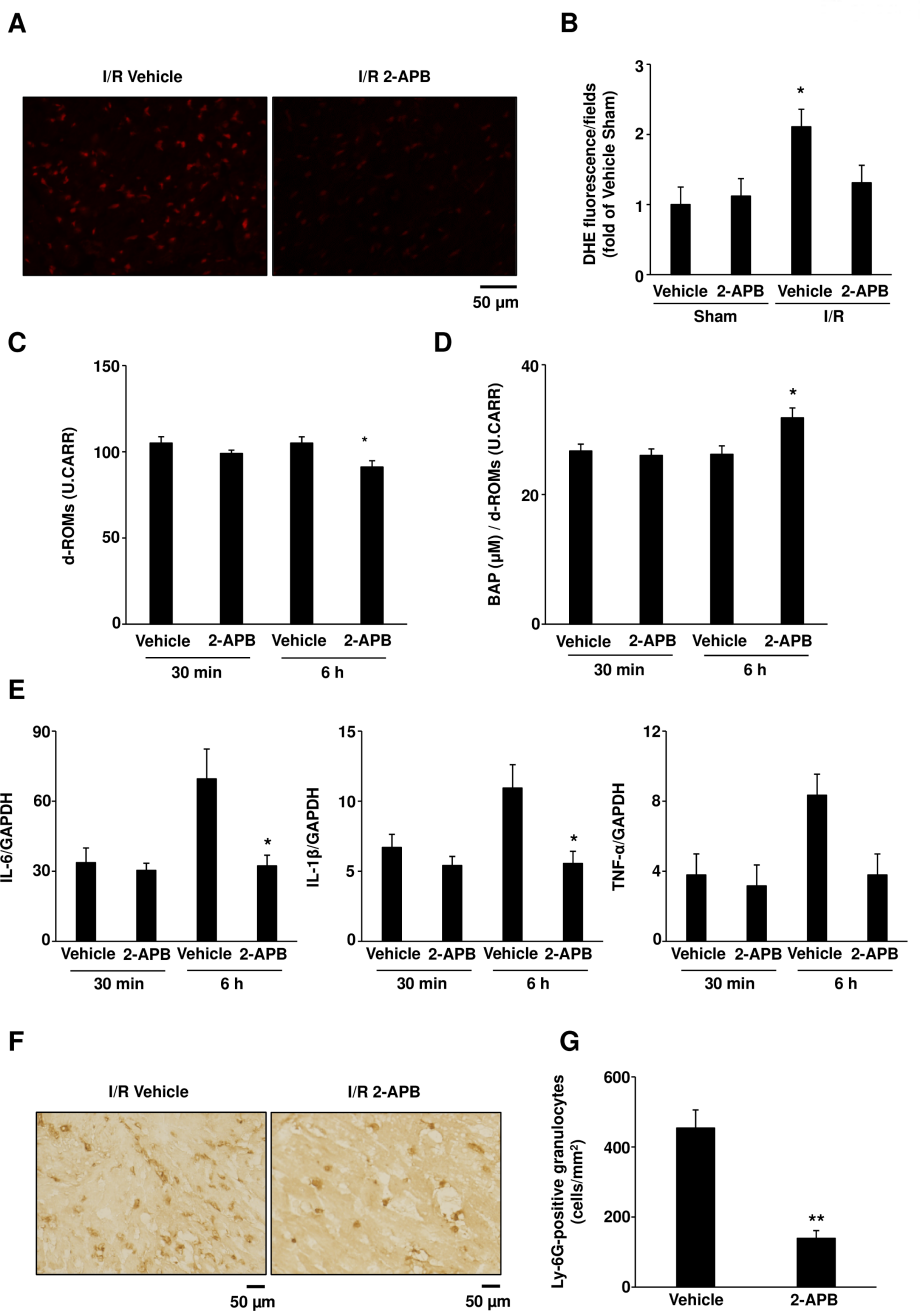
Treatment with 2-APB attenuates reactive oxygen species (ROS) production and inflammatory responses after cardiac ischemia/reperfusion (I/R) in mice. (A) Representative fluorescence images of dihydroethidium (DHE)-stained heart sections obtained at 24 h of reperfusion from the 2-APB- or vehicle-treated mice. Scale bar: 50 μm. (B) Fluorescence intensity obtained from eight images, expressed as the mean fluorescence intensity normalized to the levels obtained from mice of the vehicle sham group. Results are presented as mean values ± SEM obtained from 4–5 mice. *P<0.05 vs. I/R 2-APB-treated sample. (C and D) Effect of 2-APB on diacron reactive oxygen metabolites (d-ROMs) (C) and biological antioxidant potential (BAP)/d-ROMs (D) in mice that underwent 30 min of ischemia followed by 30 min or 6 h of reperfusion. Results are presented as mean values ± SEM obtained from 4–6 mice. 105±4 *vs*. 91±4 in U.CARR (6 h reperfusion in d-ROMs) and 26.2±1.3 *vs*. 31.8±1.5 in μM/U.CARR (6 h reperfusion in BAP/d-ROMs). *P<0.05 *vs*. I/R vehicle-treated 6 hours reperfusion group, obtained using Student’s *t*-test. (E) *Il6*, *Il1β*, and *Tnf-α* expression levels in mouse hearts. Results are presented as mean values ± SEM obtained from 7–8 mice. *P<0.05 *vs*. I/R vehicle-treated 6 h reperfusion group, obtained using Student’s *t*-test. (F) Representative images of heart sections obtained from different animal groups, stained with anti-Ly-6G antibody (Gr-1) for the detection of neutrophils. Scale bar: 50 μm. (G) Quantification of Gr-1-positive cells in ischemic myocardium presented as the number of positive cells/mm^2^. Results are presented as mean values ± SEM obtained from 6–7 mice; 454±52 cells/mm^2^ *vs*. 140±22 cells/mm^2^ for Ly-6G-positive granulocytes. **P<0.01 *vs*. I/R vehicle-treated group, obtained using Student’s *t*-test.

To investigate the functional role of ROS scavenging properties of 2-APB in the reduction of I/R injury in mice, we analyzed ROS levels in the I/R hearts using DHE staining. DHE-stained I/R hearts showed distinct signals 24 h after the reperfusion, indicating ROS production (Fig 5A). Notably, treatment with 10 mg/kg 2-APB led to a decrease in signal intensity in DHE-stained I/R heart (Fig 5A), which was shown to be significantly lower when compared to the control (fold intensity, 1.32±0.11 *vs*. 2.11±0.32, respectively; Fig 5B), suggesting that ROS production was suppressed by 2-APB treatment. To examine the involvement of ROS in 2-APB induced cardioprotection, serum d-ROM and BAP levels were determined 30 min and 6 h after the reperfusion. While no significant differences in both parameters were obtained at 30 min after the I/R, d-ROM levels were shown to be significantly decreased in 2-APB treatment group, compared with those in the vehicle-treated group 6 h after the I/R (105±4 *vs*. 91±4 U.CARR in d-ROMs and 26.2±1.3 *vs*. 31.8±1.5 in μM/U.CARR in BAP/d-ROMs;.Fig 5C). Moreover, the BAP/d-ROM ratio was shown to be significantly increased in mice treated with 2-APB, in comparison to that in the vehicle-treated group (Fig 5D), suggesting an increase in the antioxidant potential in the 2-APB-treated mice.

Since the source of oxidative stress is mainly from neutrophils and not from cardiomyocyte mitochondria in the subacute phase after the I/R, we further evaluated whether 2-APB affects the inflammatory response after myocardial I/R. Levels of *il-6* and *il-1β* expression were shown to be significantly decreased in 2-APB-treated mice compared with those in the control-I/R mice at 6 h after reperfusion (Fig 5E). The expression of *tnf*-α tended to decrease in the 2-APB-treated mice as well. Consistent with this, subsequent infiltration of inflammatory cells into the ischemic myocardium was attenuated (Fig 5F) and the number of infiltrated Ly-6G-positive granulocytes was decreased significantly in the 2-APB-treated mice at 24 h after the reperfusion (Fig 5G). Collectively, these results indicate that 2-APB treatment induces reduction of ROS and attenuates inflammatory responses in subacute phase.

## Discussion

In the present study, we demonstrated that treatment with 2-APB prevents ROS-induced cardiomyocyte death by inhibiting Ca^2+^ overload caused by an extracellular Ca^2+^ influx and attenuates cardiac I/R injury in mice. Furthermore, we demonstrated that the protective effects in cardiomyocytes are based on a direct extracellular ROS scavenging by 2-APB, rather than the channel inhibitory effects or intracellular ROS chelating. To the best of our knowledge, this study is the first to demonstrate that the antioxidative effects of 2-APB induce cardioprotection.

We demonstrated that H_2_O_2_ treatment leads to an increase intracellular Ca^2+^ levels in NRCMs consistent with previous reports [42]. The increase in Ca^2+^ was prevented by the removal of extracellular Ca^2+^, suggesting that H_2_O_2_ treatment induces Ca^2+^ influx through unknown plasma membrane ion channels. Many studies have focused on the mechanisms underlying H_2_O_2_-induced cardiomyocyte death, involving both apoptosis and necrosis [3, 43–46], and while apoptotic mechanisms have been determined [47, 48], mechanisms underlying H_2_O_2-_induced necrosis remain unknown. Our results demonstrate that H_2_O_2_-induced Ca^2+^ influx plays a major role in cardiomyocytes, and it may represent myocyte necrosis inducer.

The results of several studies showed that 2-APB protects tissues against stress [23–25, 49–51]. In liver, pretreatment with 2-APB was shown to attenuate drug-induced cellular damage and the I/R injury in mice [23, 49, 50]. Similarly, in kidneys, 2-APB was shown to exert cytoprotection after I/R by TRPM2 inhibition [24, 51]. In the heart, perfusion with 2-APB improved cardiac function on reperfusion in a rabbit *ex vivo* I/R model [52]. Here, we showed that 2-APB treatment attenuates H_2_O_2-_induced cell death by inhibiting intracellular Ca^2+^ influx. Previously, all beneficial effects of 2-APB were considered to stem from channel inhibition, however, we showed that blocking of those channels did not prevent H_2_O_2_-induced cell death. These results indicate that major 2-APB-sensitive Ca^2+^ channels are not involved in the H_2_O_2-_induced cardiomyocyte death.

We used ^1^H-NMR and TLC analysis to demonstrate that 2-APB chemically reacts with H_2_O_2_ and generates phenol, thereby exhibiting an antioxidant effect. We suggest the following process underlying the observed effects: 2-APB is oxidized by H_2_O_2_, generating a boronic ester that is hydrolyzed to produce phenol. This reaction was shown to occur within 5 min following the addition of H_2_O_2_ to 2-APB, indicating that 2-APB is highly reactive towards H_2_O_2_ and may represent a powerful antioxidant. To the best of our knowledge, this is the first evidence demonstrating a direct antioxidative effect of 2-APB. More than 800 studies using 2-APB have been published, but this compound has been, generally, used as a channel blocking reagent. Since our results indicate that 2-APB has strong antioxidative properties, we believe that previously reported results must be interpreted with caution, especially if H_2_O_2_ was used simultaneously.

While ROS plays central roles in the development of I/R injuries, the efficacy of antioxidant therapy remains controversial [53]. A single injection of NAC during ischemia failed to attenuate cardiac I/R injury [54], similar to the results obtained using our cardiac I/R model. ROS generation can affect I/R injury through two processes. First, during the acute phase of I/R injury, ROS efflux from mitochondria occurs immediately after the reperfusion in cardiomyocytes and induces cell death by triggering MPTP opening [2]. Second, ROS generated by endothelial or infiltrated inflammatory cells during the I/R extends the injury by activation of inflammatory responses [6]. Following the stimulation with proinflammatory mediators, neutrophils are recruited to the site of injury, where they produce superoxide anions through the NADPH oxidase pathway, releasing ROS to damage cells [55]. The obtained results suggest that the latter mechanism represents the target of 2-APB, resulting in ROS scavenging and cardioprotection, since 2-APB failed to inhibit intracellular ROS increase caused by PE stimulation, where ROS is produced in cardiomyocytes through membrane NAPDH activation [40, 56]. These results indicate that 2-APB treatment induces not intracellular but mainly extracellular ROS inhibition, generated from inflammatory cells during I/R injury in mice. ROS levels were shown to be significantly lower in the later phase of the I/R injury development in 2-APB treated group. Since the mitochondrial ROS efflux occurs immediately after reperfusion, those results suggest that the 2-APB target is not mitochondrial ROS efflux. The results also indicate that 2-APB treatment modulates ROS levels from the infiltrated immune cells which could be the source of ROS in the later phase. The unique properties of this compound may be more suitable for cardioprotective use than previously reported antioxidants.

This study has several limitations. First, we administered 2-APB during ischemia, immediately before the reperfusion. We selected this, since this protocol simulates the clinical use of drugs during primary coronary interventions, as it is the earliest available pharmacological intervention point, however, it may explain why no differences were observed in ROS levels after 2-APB administration immediately after the reperfusion. Furthermore, we cannot exclude the possibility that 2-APB directly inhibits inflammatory cell channels, attenuating the inflammation[57, 58]. As previously reported, 2-APB treatment may affect neutrophil activity or inhibit inflammation in nasal polyps [59, 60].

There are several reports regarding clinical trials with antioxidants for cardiac ischemia-reperfusion injury in acute coronary syndrome [61]. However, in terms of Jadad scale [62], only a few articles are of good quality. Among them, there were two clinical trials of NAC, those which were conducted using well-designed protocols [63, 64]. However, they showed controversial results. One of the major differences between 2-APB and NAC is the absence of chelation of ROS produced inside cells. This might be an advantage of 2-APB, since ROS signaling plays an important role in cellular functions, as well.

In summary, we demonstrated that 2-APB possesses antioxidative properties and the treatment with 2-APB attenuates cardiac I/R injury in mice. Furthermore, we demonstrated that the extracellular ROS mediates Ca^2+^ influx, inducing cardiomyocyte death, which can be prevented by 2-APB treatment. While the mechanisms underlying cardiac I/R injury have been intensely investigated, no pharmacological interventions that can alleviate the I/R injury are in clinical use. Our results suggest that the treatment with 2-APB is a promising novel therapeutic approach to the treatment of the cardiac I/R injury, at least partially by reducing ROS levels during subacute phase. Finally, the identification of channels responsible for H_2_O_2_-induced Ca^2+^ influxes is needed to develop novel therapeutics for improved cardioprotection.

## Supporting information

S1 Fig *N*-acetylcysteine treatment inhibited intracellular ROS increases after phenylephrine stimulation. NRCMs were loaded with 5 μM DCF and treated with *N*-acetylcysteine (NAC) for 1 hour, followed by stimulation with 2 mM phenylephrine (PE). Intracellular ROS levels are calculated as ROS increase ratio from DCF fluorescent intensity increase ratios normalized to those at 0 minute. The results of average value obtained from 3 independent experiments are depicted.
(TIF)

S2 Fig *N*-acetylcysteine treatment significantly inhibited intracellular ROS increases in a dose-dependent manner. Intracellular ROS levels at the time point of 60 minutes after PE stimulation were estimated from the measurement of DCF fluorescence in NRCMs pretreated with or without NAC (100 or 500 μM) for 1 hour. Values are shown as mean ± SEM of 3 independent experiments. **P<0.01 vs. non-treatment, ^††^*P*<0.01 vs. 2 mM PE treatment, by one-way ANOVA followed by Tukey-Kramer test.
(TIF)

S3 Fig Single administration of NAC failed to affect areas at risk in cardiac I/R injury mouse model. C57BL/6 mice were exposed to 30 minutes ischemia, followed by 24 hours reperfusion. NAC (100 mg/kg) or vehicle was administered intravenously immediate before reperfusion. Areas at risk (AAR) were estimated by exclusion of area stained with Evans blue. The myocardial infarct areas were detected by staining with 2% triphenyl tetrazolium chloride (TTC). The ratio of AAR normalized to LV was quantitatively estimated.
(TIF)

S4 Fig Single administration of NAC failed to attenuate cardiac I/R injury in mice. Size of infarct area (IA) normalized to AAR was quantitatively assessed. Values are shown as mean ± SEM (49.9±14.1% vs 41.3±9.2% in IA/AAR, n = 3 mice for vehicle; n = 5 mice for 100 mg/kg of NAC).
(TIF)

S1 Table PCR primers used in the present study. The sequences of the primers used in the present study are shown.
(PDF)

## References

[1] Yellon DM, Hausenloy DJ. Myocardial reperfusion injury. N Engl J Med. 2007;357(11):1121–35. doi: 10.1056/NEJMra071667 . 17855673 10.1056/NEJMra071667

[2] Murphy E, Steenbergen C. Mechanisms underlying acute protection from cardiac ischemia-reperfusion injury. Physiol Rev. 2008;88(2):581–609. doi: 10.1152/physrev.00024.2007 ; PubMed Central PMCID: PMCPMC3199571. 18391174 10.1152/physrev.00024.2007 PMC3199571

[3] Kalogeris T, Baines CP, Krenz M, Korthuis RJ. Cell biology of ischemia/reperfusion injury. Int Rev Cell Mol Biol. 2012;298:229–317. doi: 10.1016/B978-0-12-394309-5.00006-7 ; PubMed Central PMCID: PMCPMC3904795. 22878108 10.1016/B978-0-12-394309-5.00006-7 PMC3904795

[4] Ibanez B, Heusch G, Ovize M, Van de Werf F. Evolving therapies for myocardial ischemia/reperfusion injury. J Am Coll Cardiol. 2015;65(14):1454–71. doi: 10.1016/j.jacc.2015.02.032 . 25857912 10.1016/j.jacc.2015.02.032

[5] Garcia-Dorado D, Ruiz-Meana M, Inserte J, Rodriguez-Sinovas A, Piper HM. Calcium-mediated cell death during myocardial reperfusion. Cardiovasc Res. 2012;94(2):168–80. doi: 10.1093/cvr/cvs116 . 22499772 10.1093/cvr/cvs116

[6] Granger DN, Kvietys PR. Reperfusion injury and reactive oxygen species: The evolution of a concept. Redox Biol. 2015;6:524–51. doi: 10.1016/j.redox.2015.08.020 ; PubMed Central PMCID: PMCPMC4625011. 26484802 10.1016/j.redox.2015.08.020 PMC4625011

[7] Zweier JL, Flaherty JT, Weisfeldt ML. Direct measurement of free radical generation following reperfusion of ischemic myocardium. Proc Natl Acad Sci U S A. 1987;84(5):1404–7. ; PubMed Central PMCID: PMCPMC304438. 3029779 10.1073/pnas.84.5.1404 PMC304438

[8] Kevin LG, Camara AK, Riess ML, Novalija E, Stowe DF. Ischemic preconditioning alters real-time measure of O2 radicals in intact hearts with ischemia and reperfusion. Am J Physiol Heart Circ Physiol. 2003;284(2):H566–74. Epub 2002/11/05. doi: 10.1152/ajpheart.00711.2002 . 12414448 10.1152/ajpheart.00711.2002

[9] Yan Y, Wei CL, Zhang WR, Cheng HP, Liu J. Cross-talk between calcium and reactive oxygen species signaling. Acta Pharmacol Sin. 2006;27(7):821–6. doi: 10.1111/j.1745-7254.2006.00390.x . 16787564 10.1111/j.1745-7254.2006.00390.x

[10] Pell VR, Chouchani ET, Murphy MP, Brookes PS, Krieg T. Moving Forwards by Blocking Back-Flow: The Yin and Yang of MI Therapy. Circ Res. 2016;118(5):898–906. doi: 10.1161/CIRCRESAHA.115.306569 ; PubMed Central PMCID: PMCPMC4809200. 26941425 10.1161/CIRCRESAHA.115.306569 PMC4809200

[11] Kurian GA, Rajagopal R, Vedantham S, Rajesh M. The Role of Oxidative Stress in Myocardial Ischemia and Reperfusion Injury and Remodeling: Revisited. Oxid Med Cell Longev. 2016;2016:1656450 doi: 10.1155/2016/1656450 ; PubMed Central PMCID: PMCPMC4897712. 27313825 10.1155/2016/1656450 PMC4897712

[12] Burgoyne JR, Mongue-Din H, Eaton P, Shah AM. Redox signaling in cardiac physiology and pathology. Circ Res. 2012;111(8):1091–106. doi: 10.1161/CIRCRESAHA.111.255216 . 23023511 10.1161/CIRCRESAHA.111.255216

[13] Ma HT, Patterson RL, van Rossum DB, Birnbaumer L, Mikoshiba K, Gill DL. Requirement of the inositol trisphosphate receptor for activation of store-operated Ca2+ channels. Science. 2000;287(5458):1647–51. . 10698739 10.1126/science.287.5458.1647

[14] Bilmen JG, Michelangeli F. Inhibition of the type 1 inositol 1,4,5-trisphosphate receptor by 2-aminoethoxydiphenylborate. Cell Signal. 2002;14(11):955–60. . 12220621 10.1016/s0898-6568(02)00042-6

[15] Ma KT GB, Yang YQ, Nuttall AL, Jiang ZG. 2-Aminoethoxydiphenyl borate blocks electrical coupling and inhibits voltage-gated K+ channels in guinea pig arteriole cells. Am J Physiol Heart Circ Physiol 300(1):H335–46. 2011 doi: 10.1152/ajpheart.00737.2010 21037232 10.1152/ajpheart.00737.2010 PMC3023242

[16] Lemonnier L, Prevarskaya N, Mazurier J, Shuba Y, Skryma R. 2-APB inhibits volume-regulated anion channels independently from intracellular calcium signaling modulation. FEBS Lett. 2004;556(1–3):121–6. . 14706838 10.1016/s0014-5793(03)01387-5

[17] Ma HT, Venkatachalam K, Li HS, Montell C, Kurosaki T, Patterson RL, et al Assessment of the role of the inositol 1,4,5-trisphosphate receptor in the activation of transient receptor potential channels and store-operated Ca2+ entry channels. J Biol Chem. 2001;276(22):18888–96. doi: 10.1074/jbc.M100944200 . 11259416 10.1074/jbc.M100944200

[18] Schindl R, Kahr H, Graz I, Groschner K, Romanin C. Store depletion-activated CaT1 currents in rat basophilic leukemia mast cells are inhibited by 2-aminoethoxydiphenyl borate. Evidence for a regulatory component that controls activation of both CaT1 and CRAC (Ca(2+) release-activated Ca(2+) channel) channels. J Biol Chem. 2002;277(30):26950–8. doi: 10.1074/jbc.M203700200 . 12011062 10.1074/jbc.M203700200

[19] Xu SZ, Zeng F, Boulay G, Grimm C, Harteneck C, Beech DJ. Block of TRPC5 channels by 2-aminoethoxydiphenyl borate: a differential, extracellular and voltage-dependent effect. Br J Pharmacol. 2005;145(4):405–14. doi: 10.1038/sj.bjp.0706197 ; PubMed Central PMCID: PMCPMC1576154. 15806115 10.1038/sj.bjp.0706197 PMC1576154

[20] Li M, Jiang J, Yue L. Functional characterization of homo- and heteromeric channel kinases TRPM6 and TRPM7. J Gen Physiol. 2006;127(5):525–37. doi: 10.1085/jgp.200609502 ; PubMed Central PMCID: PMCPMC2151519. 16636202 10.1085/jgp.200609502 PMC2151519

[21] Togashi K, Inada H, Tominaga M. Inhibition of the transient receptor potential cation channel TRPM2 by 2-aminoethoxydiphenyl borate (2-APB). Br J Pharmacol. 2008;153(6):1324–30. doi: 10.1038/sj.bjp.0707675 ; PubMed Central PMCID: PMCPMC2275460. 18204483 10.1038/sj.bjp.0707675 PMC2275460

[22] Callera GE, He Y, Yogi A, Montezano AC, Paravicini T, Yao G, et al Regulation of the novel Mg2+ transporter transient receptor potential melastatin 7 (TRPM7) cation channel by bradykinin in vascular smooth muscle cells. J Hypertens. 2009;27(1):155–66. . 19145781 10.1097/hjh.0b013e3283190582

[23] Nicoud IB, Knox CD, Jones CM, Anderson CD, Pierce JM, Belous AE, et al 2-APB protects against liver ischemia-reperfusion injury by reducing cellular and mitochondrial calcium uptake. Am J Physiol Gastrointest Liver Physiol. 2007;293(3):G623–30. doi: 10.1152/ajpgi.00521.2006 . 17627971 10.1152/ajpgi.00521.2006

[24] Yildar M, Aksit H, Korkut O, Ozyigit MO, Sunay B, Seyrek K. Protective effect of 2-aminoethyl diphenylborinate on acute ischemia-reperfusion injury in the rat kidney. J Surg Res. 2014;187(2):683–9. doi: 10.1016/j.jss.2013.11.009 . 24331939 10.1016/j.jss.2013.11.009

[25] Taskin MI, Hismiogullari AA, Yay A, Adali E, Gungor AC, Korkmaz GO, et al Effect of 2-aminoethoxydiphenyl borate on ischemia-reperfusion injury in a rat ovary model. Eur J Obstet Gynecol Reprod Biol. 2014;178:74–9. doi: 10.1016/j.ejogrb.2014.03.049 . 24792541 10.1016/j.ejogrb.2014.03.049

[26] Matsuo R, Morihara H, Mohri T, Murasawa S, Takewaki K, Nakayama H, et al The inhibition of N-glycosylation of glycoprotein 130 molecule abolishes STAT3 activation by IL-6 family cytokines in cultured cardiac myocytes. PLoS One. 2014;9(10):e111097 doi: 10.1371/journal.pone.0111097 ; PubMed Central PMCID: PMCPMC4207791. 25340554 10.1371/journal.pone.0111097 PMC4207791

[27] Calvo M, Villalobos C, Nunez L. Calcium imaging in neuron cell death. Methods Mol Biol. 2015;1254:73–85. Epub 2014/11/29. doi: 10.1007/978-1-4939-2152-2_6 . 25431058 10.1007/978-1-4939-2152-2_6

[28] Silverstein RM, Webster FX, Kiemle DJ, Bryce D. Spectrometric Identification of Organic Compounds, 8th Ed. Wiley, 2014;126–320.

[29] Lewis HW, Moody CJ. Experimental Organic Chemistry: Principles and Practice, Wiley Blackwell, 1989;159–173.

[30] Obana M, Miyamoto K, Murasawa S, Iwakura T, Hayama A, Yamashita T, et al Therapeutic administration of IL-11 exhibits the postconditioning effects against ischemia-reperfusion injury via STAT3 in the heart. Am J Physiol Heart Circ Physiol. 2012;303(5):H569–77. doi: 10.1152/ajpheart.00060.2012 . 22707562 10.1152/ajpheart.00060.2012

[31] Fukui T, Yamauchi K, Maruyama M, Yasuda T, Kohno M, Abe Y. Significance of measuring oxidative stress in lifestyle-related diseases from the viewpoint of correlation between d-ROMs and BAP in Japanese subjects. Hypertens Res. 2011;34(9):1041–5. doi: 10.1038/hr.2011.76 . 21677660 10.1038/hr.2011.76

[32] Takada M, Noguchi A, Sayama Y, Kurohane Kaneko Y, Ishikawa T. Inositol 1,4,5-trisphosphate receptor-mediated initial Ca(2+) mobilization constitutes a triggering signal for hydrogen peroxide-induced apoptosis in INS-1 beta-cells. Biol Pharm Bull. 2011;34(7):954–8. . 21719997 10.1248/bpb.34.954

[33] Maruyama T, Kanaji T, Nakade S, Kanno T, Mikoshiba K. 2APB, 2-aminoethoxydiphenyl borate, a membrane-penetrable modulator of Ins(1,4,5)P3-induced Ca2+ release. J Biochem. 1997;122(3):498–505. . 9348075 10.1093/oxfordjournals.jbchem.a021780

[34] Dobrydneva Y, Blackmore P. 2-Aminoethoxydiphenyl borate directly inhibits store-operated calcium entry channels in human platelets. Mol Pharmacol. 2001;60(3):541–52. . 11502886

[35] Bootman MD, Collins TJ, Mackenzie L, Roderick HL, Berridge MJ, Peppiatt CM. 2-aminoethoxydiphenyl borate (2-APB) is a reliable blocker of store-operated Ca2+ entry but an inconsistent inhibitor of InsP3-induced Ca2+ release. FASEB J. 2002;16(10):1145–50. doi: 10.1096/fj.02-0037rev . 12153982 10.1096/fj.02-0037rev

[36] Gao H, Wang F, Wang W, Makarewich CA, Zhang H, Kubo H, et al Ca(2+) influx through L-type Ca(2+) channels and transient receptor potential channels activates pathological hypertrophy signaling. J Mol Cell Cardiol. 2012;53(5):657–67. doi: 10.1016/j.yjmcc.2012.08.005 ; PubMed Central PMCID: PMCPMC3472041. 22921230 10.1016/j.yjmcc.2012.08.005 PMC3472041

[37] Siddiqui T, Lively S, Ferreira R, Wong R, Schlichter LC. Expression and contributions of TRPM7 and KCa2.3/SK3 channels to the increased migration and invasion of microglia in anti-inflammatory activation states. PLoS One. 2014;9(8):e106087 doi: 10.1371/journal.pone.0106087 ; PubMed Central PMCID: PMCPMC4141841. 25148577 10.1371/journal.pone.0106087 PMC4141841

[38] Chen HC, Xie J, Zhang Z, Su LT, Yue L, Runnels LW. Blockade of TRPM7 channel activity and cell death by inhibitors of 5-lipoxygenase. PLoS One. 2010;5(6):e11161 doi: 10.1371/journal.pone.0011161 ; PubMed Central PMCID: PMCPMC2887440. 20567598 10.1371/journal.pone.0011161 PMC2887440

[39] Klose C, Straub I, Riehle M, Ranta F, Krautwurst D, Ullrich S, et al Fenamates as TRP channel blockers: mefenamic acid selectively blocks TRPM3. Br J Pharmacol. 2011;162(8):1757–69. doi: 10.1111/j.1476-5381.2010.01186.x ; PubMed Central PMCID: PMCPMC3081119. 21198543 10.1111/j.1476-5381.2010.01186.x PMC3081119

[40] Hahn NE, Musters RJ, Fritz JM, Pagano PJ, Vonk AB, Paulus WJ, et al Early NADPH oxidase-2 activation is crucial in phenylephrine-induced hypertrophy of H9c2 cells. Cell Signal. 2014;26(9):1818–24. doi: 10.1016/j.cellsig.2014.04.018 ; PubMed Central PMCID: PMCPMC4406486. 24794531 10.1016/j.cellsig.2014.04.018 PMC4406486

[41] Hirotani S, Otsu K, Nishida K, Higuchi Y, Morita T, Nakayama H, et al Involvement of nuclear factor-kappaB and apoptosis signal-regulating kinase 1 in G-protein-coupled receptor agonist-induced cardiomyocyte hypertrophy. Circulation. 2002;105(4):509–15. . 11815436 10.1161/hc0402.102863

[42] Yang KT, Chang WL, Yang PC, Chien CL, Lai MS, Su MJ, et al Activation of the transient receptor potential M2 channel and poly(ADP-ribose) polymerase is involved in oxidative stress-induced cardiomyocyte death. Cell Death Differ. 2006;13(10):1815–26. doi: 10.1038/sj.cdd.4401813 . 16294211 10.1038/sj.cdd.4401813

[43] Aikawa R, Nawano M, Gu Y, Katagiri H, Asano T, Zhu W, et al Insulin prevents cardiomyocytes from oxidative stress-induced apoptosis through activation of PI3 kinase/Akt. Circulation. 2000;102(23):2873–9. . 11104747 10.1161/01.cir.102.23.2873

[44] Akao M, Ohler A, O'Rourke B, Marban E. Mitochondrial ATP-sensitive potassium channels inhibit apoptosis induced by oxidative stress in cardiac cells. Circ Res. 2001;88(12):1267–75. . 11420303 10.1161/hh1201.092094

[45] Ali MA, Kandasamy AD, Fan X, Schulz R. Hydrogen peroxide-induced necrotic cell death in cardiomyocytes is independent of matrix metalloproteinase-2. Toxicol In Vitro. 2013;27(6):1686–92. doi: 10.1016/j.tiv.2013.04.013 . 23665313 10.1016/j.tiv.2013.04.013

[46] Marshall KD, Edwards MA, Krenz M, Davis JW, Baines CP. Proteomic mapping of proteins released during necrosis and apoptosis from cultured neonatal cardiac myocytes. Am J Physiol Cell Physiol. 2014;306(7):C639–47. doi: 10.1152/ajpcell.00167.2013 ; PubMed Central PMCID: PMCPMC3962598. 24401845 10.1152/ajpcell.00167.2013 PMC3962598

[47] Crow MT, Mani K, Nam YJ, Kitsis RN. The mitochondrial death pathway and cardiac myocyte apoptosis. Circ Res. 2004;95(10):957–70. doi: 10.1161/01.RES.0000148632.35500.d9 . 15539639 10.1161/01.RES.0000148632.35500.d9

[48] Whelan RS, Kaplinskiy V, Kitsis RN. Cell death in the pathogenesis of heart disease: mechanisms and significance. Annu Rev Physiol. 2010;72:19–44. doi: 10.1146/annurev.physiol.010908.163111 . 20148665 10.1146/annurev.physiol.010908.163111 PMC12973270

[49] Patel SJ, Milwid JM, King KR, Bohr S, Iracheta-Vellve A, Li M, et al Gap junction inhibition prevents drug-induced liver toxicity and fulminant hepatic failure. Nat Biotechnol. 2012;30(2):179–83. doi: 10.1038/nbt.2089 ; PubMed Central PMCID: PMCPMC3609650. 22252509 10.1038/nbt.2089 PMC3609650

[50] Du K, Williams CD, McGill MR, Xie Y, Farhood A, Vinken M, et al The gap junction inhibitor 2-aminoethoxy-diphenyl-borate protects against acetaminophen hepatotoxicity by inhibiting cytochrome P450 enzymes and c-jun N-terminal kinase activation. Toxicol Appl Pharmacol. 2013;273(3):484–91. doi: 10.1016/j.taap.2013.09.010 ; PubMed Central PMCID: PMCPMC3858533. 24070586 10.1016/j.taap.2013.09.010 PMC3858533

[51] Gao G, Wang W, Tadagavadi RK, Briley NE, Love MI, Miller BA, et al TRPM2 mediates ischemic kidney injury and oxidant stress through RAC1. J Clin Invest. 2014;124(11):4989–5001. doi: 10.1172/JCI76042 ; PubMed Central PMCID: PMCPMC4347231. 25295536 10.1172/JCI76042 PMC4347231

[52] Gysembergh A, Lemaire S, Piot C, Sportouch C, Richard S, Kloner RA, et al Pharmacological manipulation of Ins(1,4,5)P3 signaling mimics preconditioning in rabbit heart. Am J Physiol. 1999;277(6 Pt 2):H2458–69. . 10600869 10.1152/ajpheart.1999.277.6.H2458

[53] Hausenloy DJ, Yellon DM. Myocardial ischemia-reperfusion injury: a neglected therapeutic target. J Clin Invest. 2013;123(1):92–100. doi: 10.1172/JCI62874 ; PubMed Central PMCID: PMCPMC3533275. 23281415 10.1172/JCI62874 PMC3533275

[54] Abe M, Takiguchi Y, Ichimaru S, Tsuchiya K, Wada K. Comparison of the protective effect of N-acetylcysteine by different treatments on rat myocardial ischemia-reperfusion injury. J Pharmacol Sci. 2008;106(4):571–7. . 18385540 10.1254/jphs.fp0071664

[55] El-Benna J, Dang PM, Gougerot-Pocidalo MA. Priming of the neutrophil NADPH oxidase activation: role of p47phox phosphorylation and NOX2 mobilization to the plasma membrane. Semin Immunopathol. 2008;30(3):279–89. doi: 10.1007/s00281-008-0118-3 . 18536919 10.1007/s00281-008-0118-3

[56] Tanaka K, Honda M, Takabatake T. Redox regulation of MAPK pathways and cardiac hypertrophy in adult rat cardiac myocyte. J Am Coll Cardiol. 2001;37(2):676–85. . 11216996 10.1016/s0735-1097(00)01123-2

[57] Itagaki K, Kannan KB, Singh BB, Hauser CJ. Cytoskeletal reorganization internalizes multiple transient receptor potential channels and blocks calcium entry into human neutrophils. J Immunol. 2004;172(1):601–7. Epub 2003/12/23. . 14688372 10.4049/jimmunol.172.1.601

[58] Syed Mortadza SA, Wang L, Li D, Jiang LH. TRPM2 Channel-Mediated ROS-Sensitive Ca(2+) Signaling Mechanisms in Immune Cells. 2015;6:407 Epub 2015/08/25. doi: 10.3389/fimmu.2015.00407 PubMed PMID: 26300888; PubMed Central PMCID: PMCPMC4528159. 26300888 10.3389/fimmu.2015.00407 PMC4528159

[59] Conejeros I, Velasquez ZD, Carretta MD, Alarcon P, Hidalgo MA, Burgos RA. 2-Aminoethoxydiphenyl borate (2-APB) reduces alkaline phosphatase release, CD63 expression, F-actin polymerization and chemotaxis without affecting the phagocytosis activity in bovine neutrophils. Vet Immunol Immunopathol. 2012;145(1–2):540–5. doi: 10.1016/j.vetimm.2011.12.006 . 22226550 10.1016/j.vetimm.2011.12.006

[60] Lin L, Dai F, Chen Z, Cai L. In Vitro Treatment with 2-APB Inhibits the Inflammation in Nasal Polyps. Otolaryngol Head Neck Surg. 2015;153(3):461–7. doi: 10.1177/0194599815589582 . 26084825 10.1177/0194599815589582

[61] Ekelof S, Jensen SE, Rosenberg J, Gogenur I. Reduced oxidative stress in STEMI patients treated by primary percutaneous coronary intervention and with antioxidant therapy: a systematic review. Cardiovasc Drugs Ther. 2014;28(2):173–81. Epub 2014/02/18. doi: 10.1007/s10557-014-6511-3 . 24532094 10.1007/s10557-014-6511-3

[62] Jadad AR, Moore RA, Carroll D, Jenkinson C, Reynolds DJ, Gavaghan DJ, et al Assessing the quality of reports of randomized clinical trials: is blinding necessary? Control Clin Trials. 1996;17(1):1–12. Epub 1996/02/01. . 8721797 10.1016/0197-2456(95)00134-4

[63] Thiele H, Hildebrand L, Schirdewahn C, Eitel I, Adams V, Fuernau G, et al Impact of high-dose N-acetylcysteine versus placebo on contrast-induced nephropathy and myocardial reperfusion injury in unselected patients with ST-segment elevation myocardial infarction undergoing primary percutaneous coronary intervention. The LIPSIA-N-ACC (Prospective, Single-Blind, Placebo-Controlled, Randomized Leipzig Immediate PercutaneouS Coronary Intervention Acute Myocardial Infarction N-ACC) Trial. J Am Coll Cardiol. 2010;55(20):2201–9. Epub 2010/05/15. doi: 10.1016/j.jacc.2009.08.091 . 20466200 10.1016/j.jacc.2009.08.091

[64] Pasupathy S, Tavella R, Grover S, Raman B, Procter NEK, Du YT, et al Early Use of N-acetylcysteine With Nitrate Therapy in Patients Undergoing Primary Percutaneous Coronary Intervention for ST-Segment-Elevation Myocardial Infarction Reduces Myocardial Infarct Size (the NACIAM Trial [N-acetylcysteine in Acute Myocardial Infarction]). Circulation. 2017;136(10):894–903. Epub 2017/06/22. doi: 10.1161/CIRCULATIONAHA.117.027575 . 28634219 10.1161/CIRCULATIONAHA.117.027575

