# Supplementary figures and images for: 2-aminoethoxydiphenyl borate provides an anti-oxidative effect and mediates cardioprotection during ischemia reperfusion in mice
Source: PLoS One. 2017 Dec 21;12(12):e0189948. doi: 10.1371/journal.pone.0189948 (PMC5739451; doi:10.1371/journal.pone.0189948)

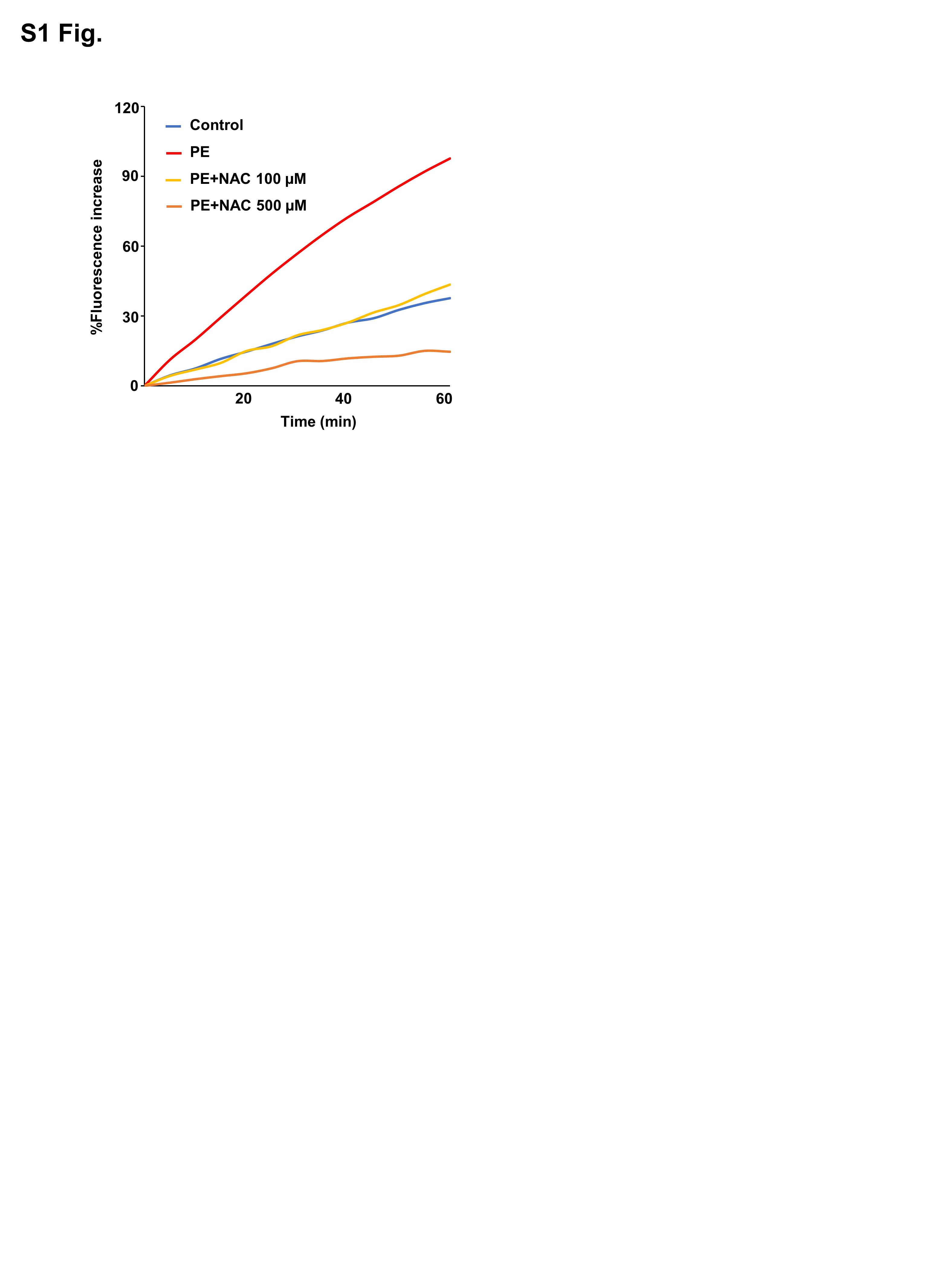

Supplement: S1 Fig — NRCMs were loaded with 5 μM DCF and treated with N-acetylcysteine (NAC) for 1 hour, followed by stimulation with 2 mM phenylephrine (PE). Intracellular ROS levels are calculated as ROS increase ratio from DCF fluorescent intensity increase ratios normalized to those at 0 minute. The results of average value obtained from 3 independent experiments are depicted. (TIF) [file pone.0189948.s001.tif]

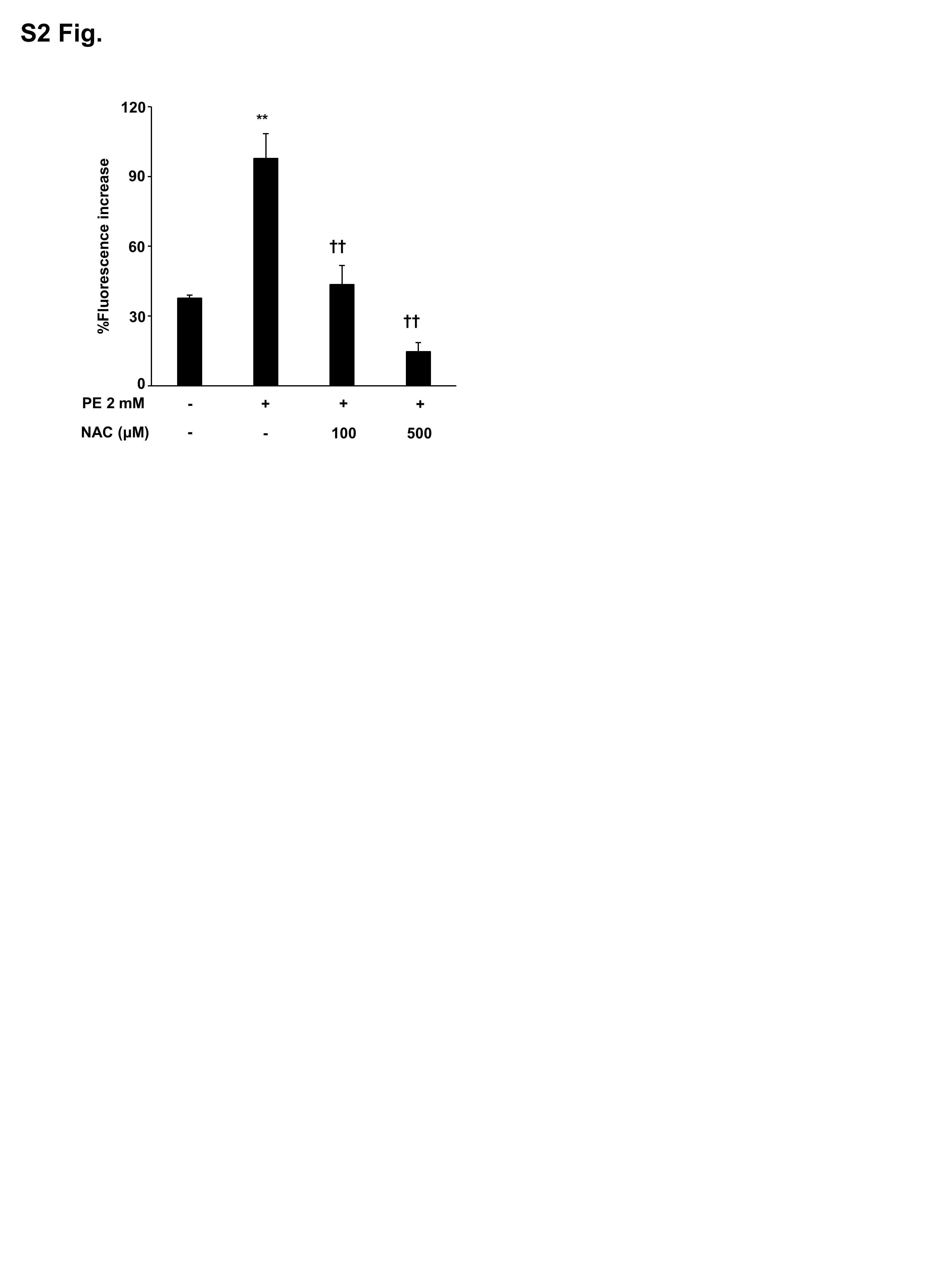

Supplement: S2 Fig — Intracellular ROS levels at the time point of 60 minutes after PE stimulation were estimated from the measurement of DCF fluorescence in NRCMs pretreated with or without NAC (100 or 500 μM) for 1 hour. Values are shown as mean ± SEM of 3 independent experiments. **P<0.01 vs. non-treatment, ††P<0.01 vs. 2 mM PE treatment, by one-way ANOVA followed by Tukey-Kramer test. (TIF) [file pone.0189948.s002.tif]

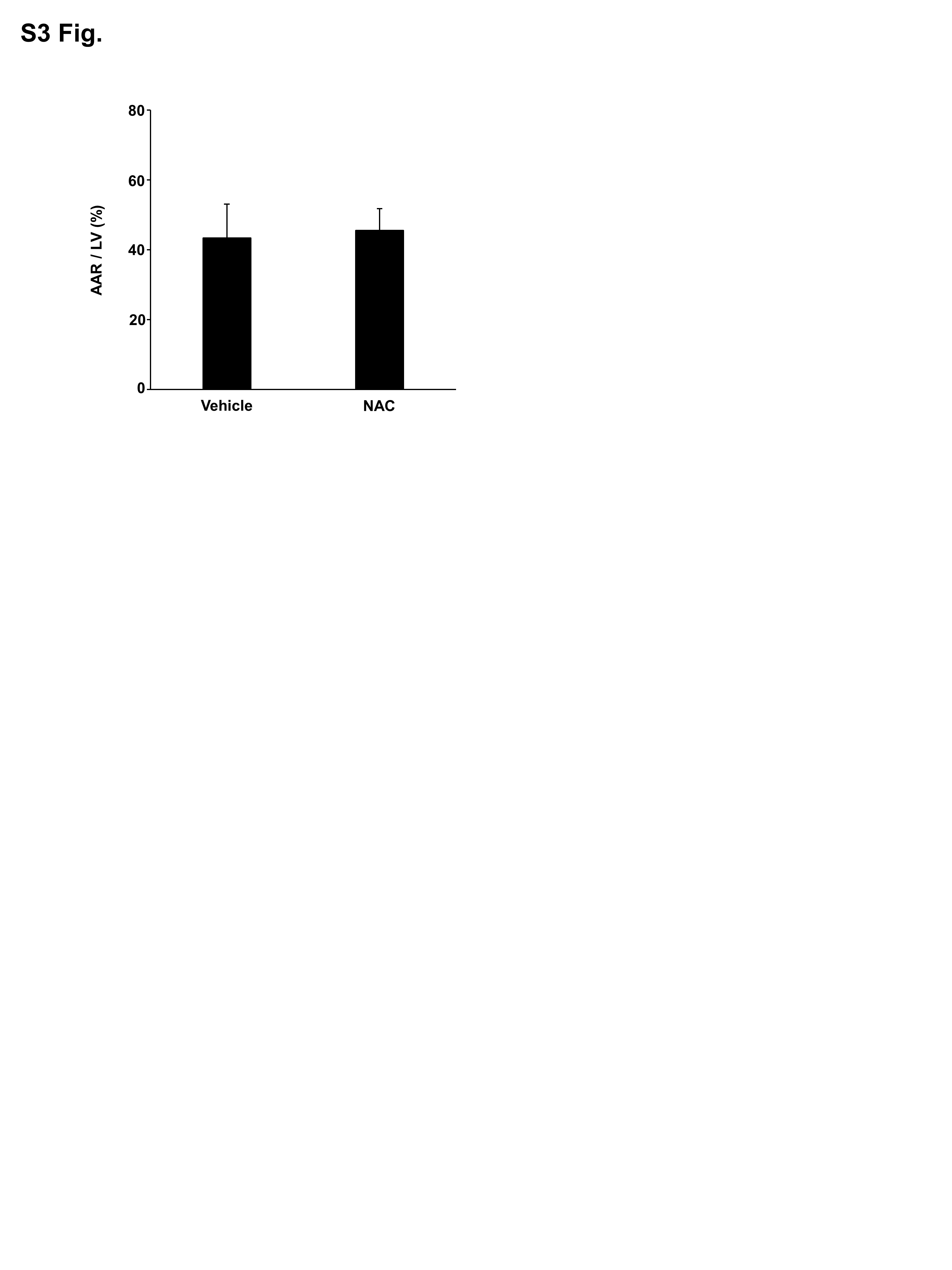

Supplement: S3 Fig — C57BL/6 mice were exposed to 30 minutes ischemia, followed by 24 hours reperfusion. NAC (100 mg/kg) or vehicle was administered intravenously immediate before reperfusion. Areas at risk (AAR) were estimated by exclusion of area stained with Evans blue. The myocardial infarct areas were detected by staining with 2% triphenyl tetrazolium chloride (TTC). The ratio of AAR normalized to LV was quantitatively estimated. (TIF) [file pone.0189948.s003.tif]

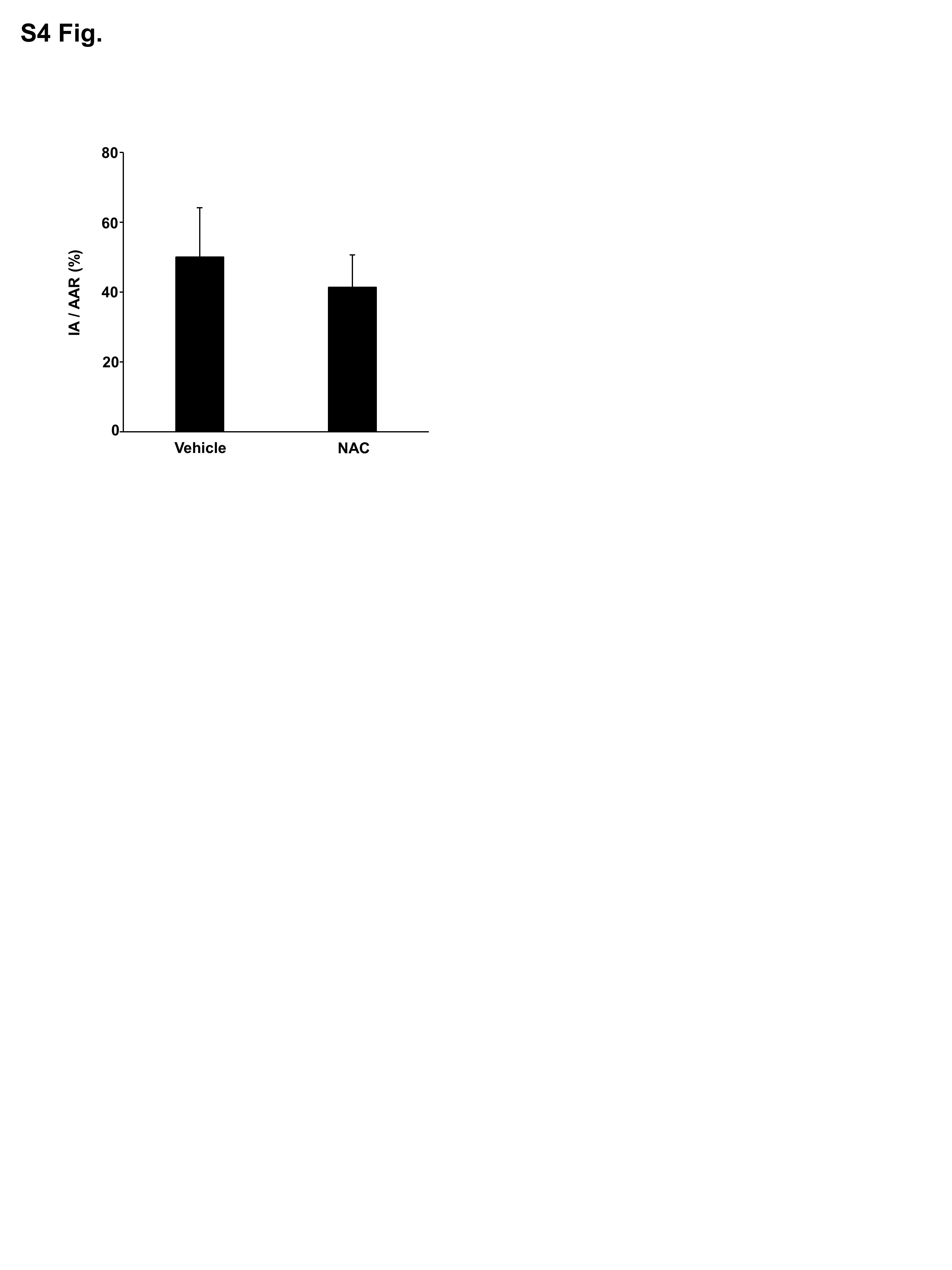

Supplement: S4 Fig — Size of infarct area (IA) normalized to AAR was quantitatively assessed. Values are shown as mean ± SEM (49.9±14.1% vs 41.3±9.2% in IA/AAR, n = 3 mice for vehicle; n = 5 mice for 100 mg/kg of NAC). (TIF) [file pone.0189948.s004.tif]
