# Supplementary material for: 2-aminoethoxydiphenyl borate provides an anti-oxidative effect and mediates cardioprotection during ischemia reperfusion in mice
Source: PLoS One. 2017 Dec 21;12(12):e0189948. doi: 10.1371/journal.pone.0189948 (PMC5739451; doi:10.1371/journal.pone.0189948)
Supplement: S1 Table — The sequences of the primers used in the present study are shown. (PDF) [file pone.0189948.s005.pdf]

Supplemental Data

Supplemental Table1. PCR primers used in the present study.

Gene	Direction	Sequence
**TRPC1**	Forward	5’- CAGACATTCCAGGTTTCGTC -3’
	Reverse	5’- AGTCCTCGTTTGTCAAGAGG -3’
**TRPC3**	Forward	5’- TCGCCATCAGCAAAGGCTAT -3’
	Reverse	5’- GCGACAGAAGTAGTCGTGTG -3’
**TRPC5**	Forward	5’- GTGTAAGCTCTTTGCCAAGG -3’
	Reverse	5’- CAGTGTTTTCTTCGCCATCC -3’
**TRPC6**	Forward	5’- AGGTCTTTATGCAATCGCGG -3’
	Reverse	5’- CCACCGACTTCACTTCAGAA -3’
**TRPC7**	Forward	5’- CTTACGGCAACAGTCTATCG -3’
	Reverse	5’- GAACTGCGTGGTTTTCACTC -3'
**TRPM2**	Forward	5’- GGATGTTGCCATTCTGCAAG -3’
	Reverse	5’- TCAGGCTTGTTGGAGATGAG -3’
**TRPM3**	Forward	5’- ACCAGACCATGTCAAACCCT -3’
	Reverse	5’- AGGGGTGTCTCGAAGGTATT -3’
**TRPM7**	Forward	5’- GGGTAATCTTCCTCCAGGGT -3’
	Reverse	5’- GTCTGTAGGGTTGGGCTGTT -3’
**IL-6**	Forward	5’- AAGAGACTTCCATCCAGTTGCCTTC -3’
	Reverse	5’- ATTATATCCAGTTTGGTAGCATCCATC -3’
**IL-1β**	Forward	5’- GCAGCTATGGCAACTGTTCCT -3’
	Reverse	5’- ATGAGTGTAACTGCCTGCCTG -3’
**TNF-α**	Forward	5’- CAAAACTTCGAGTGACAAGCCTGTAGC -3’
	Reverse	5’- CCCTTGAAGAGAACCTGGGAGTAGA -3’
**GAPDH**	Forward	5’- CATCACCATCTTCCAGGAGCG -3’
	Reverse	5’- GAGGGGCCATCCACAGTCTTC -3’
